# Measuring capacity to use evidence-based interventions in community-based organizations: A comprehensive, scoping review

**DOI:** 10.1017/cts.2022.426

**Published:** 2022-07-11

**Authors:** Shoba Ramanadhan, Sitara L. Mahtani, Shinelle Kirk, Michelle Lee, Maggie Weese, Carol Mita, Heather M. Brandt

**Affiliations:** 1 Harvard T.H. Chan School of Public Health, Boston, MA, USA; 2 Dana-Farber Cancer Institute, Boston, MA, USA; 3 Boston Medical Center, Boston, MA, USA; 4 University of California San Francisco, San Francisco, CA, USA; 5 Countway Library, Harvard Medical School, Boston, MA, USA; 6 St. Jude Children’s Research Hospital, Memphis, TN, USA

**Keywords:** Community-based organizations, capacity-building, measurement, evidence-based interventions, implementation science

## Abstract

**Introduction:**

Community-based organizations (CBOs) are well-positioned to incorporate research evidence, local expertise, and contextual factors to address health inequities. However, insufficient capacity limits use of evidence-based interventions (EBIs) in these settings. Capacity-building implementation strategies are popular, but a lack of standard models and validated measures hinders progress in the field. To advance the literature, we conducted a comprehensive scoping review.

**Methods:**

With a reference librarian, we executed a comprehensive search strategy of PubMed/Medline, Web of Science Core Collection, and EBSCO Global Health. We included articles that addressed implementation science, capacity-building, and CBOs. Of 5527 articles, 99 met our inclusion criteria, and we extracted data using a double-coding process

**Results:**

Of the 99 articles, 47% defined capacity explicitly, 31% defined it indirectly, and 21% did not define it. Common concepts in definitions were skills, knowledge/expertise, and resources. Of the 57 articles with quantitative analysis, 48 (82%) measured capacity, and 11 (23%) offered psychometric data for the capacity measures. Of the 99 studies, 40% focused exclusively on populations experiencing inequities and 22% included those populations to some extent. The bulk of the studies came from high-income countries.

**Conclusions:**

Implementation scientists should 1) be explicit about models and definitions of capacity and strategies for building capacity, 2) specify expected multi-level implementation outcomes, 3) develop and use validated measures for quantitative work, and 4) integrate equity considerations into the conceptualization and measurement of capacity-building efforts. With these refinements, we can ensure that the necessary supports reach CBO practitioners and critical partners for addressing health inequities.

## Introduction

As trusted local actors, community-based organizations (CBOs) are well-positioned to incorporate research evidence, local expertise, and contextual factors to improve health [1–4]. These organizations often fill important gaps in reaching populations served ineffectively by traditional healthcare channels and offer a unique opportunity to promote health equity [4,5]. The scale of their potential impact is substantial – CBOs delivered about $200 billion in services in the US in 2017 [6]. The term CBOs refers to mission-driven organizations that address community needs and reflect community values, are typically nonprofit and led by a board of members, and deliver services in coordination with community stakeholders [7]. While CBOs can be core implementation channels for evidence-based interventions (EBIs), they face several challenges in this regard. Barriers include insufficient training and skills to use EBIs, competing priorities, balancing capacity-building and service delivery, insufficient organizational supports for the use of EBIs, and a lack of clarity around how to sustain successful EBIs [8–12]. These challenges are particularly relevant for CBOs working with communities that have been and/or are currently being marginalized and excluded from opportunities for health and wellbeing, where resource constraints are often heightened [5,9]. Building capacity for EBI use is a critical element of designing for dissemination and implementation, for example, as highlighted by Interactive Systems Framework and the push-pull-capacity model [13,14]. Capacity to use EBIs is a driver of implementation outcomes and, ultimately, health impact and is thus a critical area of focus [10]. Capacity-building to implement EBIs has attracted a fair amount of attention, with successes in increasing the adoption and implementation of EBIs, for example, among the staff of local health departments, policymakers, and some community-based settings [15,16,10].

It is difficult to capitalize on the capacity-building literature given a lack of consensus regarding the definition of capacity as a concept. The World Health Organization describes capacity as the “knowledge, skills, commitment, structures, systems, and leadership to enable effective health promotion” [17]. This is echoed by an influential synthesis of the literature on capacity-building for EBI use, which describes capacity as having sufficient structures, personnel, and resources to utilize EBIs [10]. Further expanding potential conceptualizations, frameworks such as the Interactive Systems Framework attend to capacity in the systems integral to putting EBIs into practice, emphasizing general capacity and EBI-specific capacity [14].

**Fig. 1.**
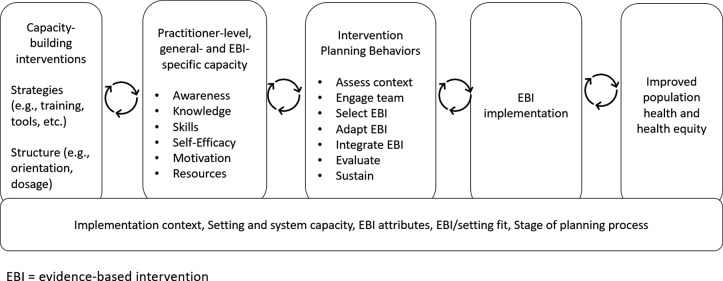
Conceptual framework for the review, adapted from Leeman and colleagues [25].

Another limitation in the field is a shortage of validated measures of capacity generally [18,19] and for use in CBOs [10]. While the use of reliable and valid measures is integral to advancing knowledge regarding the capacity-building implementation strategies that warrant further attention, most measures have been inadequately assessed for psychometric properties [10,20]. Where validated measures exist, they were often developed for non-CBO practitioners, such as health department staff, and include items that would be irrelevant in CBOs, for example, items that ask about consultations with staff epidemiologists [21]. The measurement gaps matter, as limited data describe the link between capacity-building strategies, capacity, and implementation outcomes [22]. Burgeoning efforts to bridge this measurement gap have yielded essential assessment tools to improve the implementation of EBIs in local settings [21,23]. A final potential gap in the literature relates to the need to tailor capacity-building interventions to adjust for the context in which an EBI will be implemented. On one hand, CBOs serving marginalized populations are recognized as prime partners for delivering EBIs to advance health equity [4,5]. On the other, our previous work highlights a disconnect that practitioners working with marginalized populations perceive between capacity-building interventions and their needs and expertise [24]. We were unable to find an assessment of the extent to which these organizations are present in the capacity-building literature, prompting further attention. Given the importance of increasing CBO capacity to utilize EBIs in the service of improved population health and health equity, we conducted a scoping review to examine the available literature and identify important research gaps. Our study focused on researchers addressing capacity-building for EBI use in CBOs and asked 1) how is capacity defined and conceptualized, 2) to what extent are validated measures available and used, and 3) to what extent is equity a focus in this work? The inquiry is grounded in a systematic review of capacity-building for EBI use in community settings by Leeman and colleagues, which defines capacity as the general and program-specific awareness, knowledge, skills, self-efficacy, and motivation to use an EBI. The review also identified several capacity-building strategies shown to increase adoption and implementation, such as providing technical assistance in addition to training and tools [25]. We have adapted this work to serve as the conceptual framework for this review, as summarized in Fig. 1.

## Materials and Methods

### Design

A team of researchers conducted this review. Two of the authors (SR and HMB) have been studying the use of EBIs in community settings for more than 15 years. Three members of the team were students (of public health, medicine, and psychology) (MW, ML, SK), one member manages implementation science projects (SLM), and one member (CM) is a research librarian at Harvard Medical School’s Countway Library. The team had the necessary complementary expertise to conduct the review. We did not register the scoping review given its exploratory nature. The researchers adapted the process described by Katz and Wandersman [26]. We utilized the PRISMA checklist for scoping reviews to support reporting [27] and have provided details as Supplemental File 1.

Step 1: Identify the research questions. 1) How are researchers defining and conceptualizing “capacity” and related outcomes to support the use of EBIs in CBOs? 2) To what extent are validated measures available and used? 3) To what extent are capacity-building studies attending to health equity?

Step 2: Conduct the search. Relevant studies were identified by searching the following databases: PubMed/Medline (National Library of Medicine), Web of Science Core Collection (Clarivate), and Global Health (C.A.B. International, EBSCO), on August 13, 2021. Controlled vocabulary terms (i.e., MeSH or Global Health thesaurus terms) were included when available and appropriate. The search strategies were designed and executed by a research librarian (CM). No language limits or year restrictions were applied, and bibliographies of relevant articles were reviewed to identify additional studies. We sought articles at the intersection of three core areas: 1) CBOs, 2) evidence-based practice, and 3) capacity-building. The search strategy used in PubMed included the combination of MeSH terms and keywords searched within the title and abstract was as follows:

(“Community Health Workers”[Mesh] OR “Community Health Services”[Mesh:NoExp] OR “Health Promotion”[Mesh:NoExp] OR “Organizations, Nonprofit”[Mesh:NoExp] OR “Health Education”[Mesh:NoExp] OR “Patient Education as Topic”[Mesh] OR “Consumer Health Information”[Mesh] OR community-based[tiab] OR community health[tiab] OR consumer health[tiab] OR health education[tiab] OR health promotion[tiab] OR Lady health worker*[tiab] OR Lay health worker*[tiab] OR Village health worker*[tiab] OR local organization*[tiab] OR non-clinical[tiab] OR non profit*[tiab] OR nonprofit*[tiab] OR prevention support[tiab] OR community organization*[tiab] OR “Public Health Practice”[Mesh:noexp] OR public health practic*[tiab]) **AND** (“Evidence-Based Practice”[Mesh:noexp] OR “Implementation Science”[Mesh] OR evidence based[tiab] OR evidence informed[tiab] OR effective intervention*[tiab] OR knowledge translation[tiab] OR implementation science[tiab] OR practice-based evidence [tiab]) **AND** (“Capacity Building”[Mesh] OR “Professional Competence”[Mesh:NoExp] OR “Staff Development”[Mesh] OR capacity[tiab] OR competencies[tiab] OR skills[tiab] OR work force[tiab] OR workforce[tiab] OR professional development[tiab] OR staff[tiab] OR practitioners[tiab] OR knowledge broker*[tiab]).

The search strategies for the other databases appear in Supplemental File 2. As noted elsewhere, terminology in this area has not been standardized [25]. The researchers worked with the librarian to identify a broad list of search terms to be sufficiently inclusive.

Step 3. Select articles based on the following inclusion/exclusion criteria. We imported search results into Covidence software. For each article, pairs of study team members reviewed the title and abstract. Inclusion criteria were as follows: 1) addressed CBOs AND health-focused EBIs AND capacity; 2) addressed practitioner capacity-building; 3) articles were retrievable as full-text in English. Exclusion criteria were as follows: 1) did not address the capacity of the workforce (e.g., related only to community capacity); 2) referred to capacity-building, but not in a substantive way; 3) capacity-building was explored, but not concerning EBIs; 4) article did not report on a study or conceptual model (e.g., letter to the editor). The research team reviewed and resolved conflicts at this stage as a team, with mediation by the lead author. The same process was utilized for the review of full-text articles. We included review articles to examine conceptualizations of capacity-building and to identify additional studies for inclusion.

Step 4. Extract and code data from the articles. Using Excel, pairs of researchers double-coded data for each article, and the first author resolved conflicts in the final stage. We drew on previous reviews of capacity-building to identify the fields to extract [25]. Basic study information included location (country plus state for US), conceptual vs. empirical piece, setting (e.g., CBO), types of practitioners targeted (e.g., CBO staff), health focus (e.g., obesity prevention), and extent to which the study focused on health equity. We also coded the use of qualitative and/or quantitative data. For capacity-building, we coded the level of focus and whether a definition of capacity was offered (directly, indirectly, or not at all). We coded for whether or not capacity was measured. For articles in which capacity was measured quantitatively, we assessed whether or not psychometric data were provided. Finally, we extracted the identified outcomes of capacity-building highlighted by the article.

A few categories deserve further explanation. To describe the health equity focus, the team coded presence or absence of an emphasis on at least one of the following: 1) For US studies, NIH-designated US health disparity populations as defined by NIMCHD [28], including Blacks/African Americans, Hispanics/Latinos, American Indians/Alaska Natives, Asian Americans, Native Hawaiians, and other Pacific Islanders, socioeconomically disadvantaged populations, underserved rural populations, and sexual and gender minorities; 2) other underserved populations from high-income countries (e.g., medically underserved communities, incarcerated populations, disabled populations); and 3) populations from low- and middle-income countries. For articles with a focus on these populations, we also coded whether the study included these populations (e.g., including racial and ethnic minorities as part of a general recruitment effort) or focused on them (e.g., a study that delivered a capacity-building intervention to organizations serving low-income communities).

Step 5. Analyze and summarize the data. Once the dataset was finalized, the data were summarized using descriptive statistics. All analyses were conducted using Microsoft Excel.

## Results

### Search Results

**Fig. 2.**
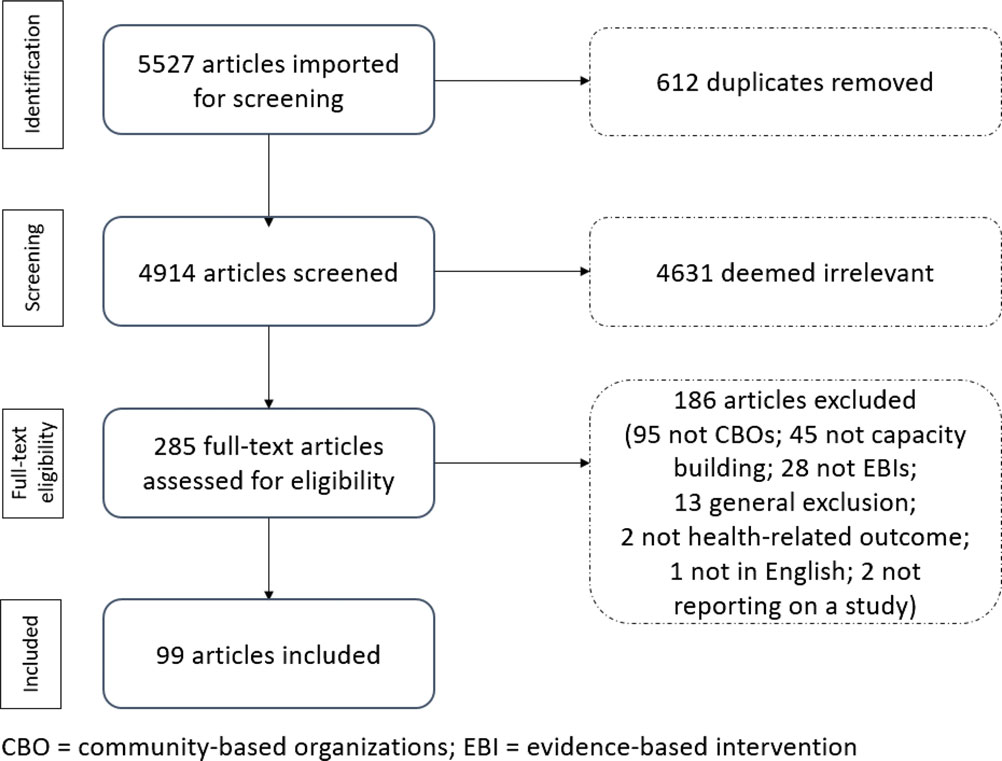
PRISMA flow chart.

As seen in Fig. 2, the initial search yielded 5527 articles, 285 full-text articles were screened, and a pool of 99 articles was retained for the review. This process is visualized according to the PRISMA reporting standards [29].

Core attributes of the 99 included articles are presented in Table 1.

**Table 1.** Description of included publications (n = 99)

Citation	Country and US State(s) if applicable	Target Practitioners	Health focus	Capacity measured	Measure psycho-metrics presented	Health equity focus (None / Close to None, Included, Primary)
Acosta et al. 2013 [30]	US - ME	Program staff	Positive youth development	Yes	Yes	None
Ai et al. 2021 [62]	US - KS	Program staff	Positive youth development	No	No	None
Allen et al. 2015 [63]	US - MA	FBO staff	Cancer control	Yes	No	Primary
Allen et al. 2016 [64]	US - MA	FBO staff	Cancer control	No	No	Primary
Allen et al. 2018 [33]	US - GA	Multiple	Chronic disease prevention	Yes	Yes	None
Allen et al. 2020 [65]	US - MA	FBO staff	Cancer control	Yes	No	Primary
Ayala et al. 2007 [66]	US - Western region	CBO staff	HIV prevention	Yes	No	Primary
Ayer et al. 2020 [67]	US - NY	Program staff	Mental health	Yes	No	Included
Bach-Mortensen et al. 2018 [9]	Multiple countries	Program staff	Multiple	No	No	None
Berman et al. 2018 [68]	US - KS, MO	Multiple	Childhood obesity	No	No	Included
Bravo et al. 2019 [69]	US - CA	Program staff	Clinical preventive services	No	No	Primary
Brock et al. 2019 [34]	US - NC, VA	Community partners	Childhood obesity	Yes	Yes	Primary
Brodowski et al. 2013 [70]	US - KS, NE	Program staff	Child abuse and neglect prevention	No	No	None
Brook & Akin 2019 [71]	US	Program staff	Multiple	No	No	Included
Brown et al. 2005 [72]	US - CA	CBO staff	STI prevention	No	No	Included
Brown et al. 2010 [73]	US - PA	Coalition members, Program staff	Risky behavior prevention in youth	Yes	No	Included
Brown et al. 2015 [74]	US - PA	Coalition members, Program staff	Crime prevention	Yes	No	Included
Brown et al. 2021 [35]	US - PA and MO	Program staff	Substance abuse prevention	Yes	Yes	None
Brownson et al. 2018 [10]	US	Program staff	Multiple	No	No	None
Bull & Dale 2021 [75]	Scotland	Program staff	General health promotion	Yes	No	Included
Cambon et al. 2017 [76]	France	Multiple	Multiple	No	No	None
Cannon et al. 2019 [77]	US - CA	Program staff	Substance abuse prevention	Yes	No	Included
Carroll-Scott et al. 2012 [78]	US - CA	CBO staff	Not specified	Yes	No	Included
Chilenski et al. 2016 [79]	US - IA, PA	Multiple	Not specified	No	No	Primary
Chilenski et al. 2018 [80]	US - IA, PA	Multiple	Youth substance abuse and problem behaviors	Yes	No	Primary
Chinman et al. 2005 [81]	US	Program staff	Substance abuse prevention	No	No	None
Chinman et al. 2008 [31]	US - CA, SC	Program staff	Substance abuse prevention	Yes	Yes	None
Chinman et al. 2012 [36]	US - ME	CBO staff	Substance abuse prevention	Yes	Yes	None
Chinman et al. 2012 [82]	US - Northeast region	Program staff	Mental health, Homelessness	No	No	Primary
Chinman et al. 2013 [83]	US - AL, GA	Program staff	STI prevention, Pregnancy prevention	Yes	No	Primary
Chinman et al. 2013 [37]	US - ME	Coalition members, Program staff	Positive youth development	Yes	Yes	None
Chinman et al. 2016 [84]	US - AL, GA	Program staff	STI prevention, Pregnancy prevention	Yes	No	Primary
Chinman et al. 2018 [85]	US - CA	Program staff	Substance abuse prevention	No	No	Primary
Claussen et al. 2017 [86]	Canada	Multiple	Domestic violence	No	No	None
Collins et al. 2006 [87]	US - Multiple	Program staff	HIV prevention	No	No	Included
Collins et al. 2007 [22]	US	CBO staff	HIV prevention	No	No	Included
Collins & Sapiano 2016 [88]	US	Program staff	HIV prevention	No	No	Included
Crowley et al. 2012 [89]	US - IA, PA	Multiple	Youth substance abuse prevention	Yes	No	Primary
Douglas et al. 2019 [90]	US - OK	Program staff	Chronic disease prevention	Yes	No	Primary
Duffy et al. 2012 [91]	US - SC	Program staff	Pregnancy prevention	Yes	No	None
Escoffery et al. 2012 [92]	US - GA	Multiple	Chronic disease prevention, Cancer control	Yes	No	None
Escoffery et al. 2015 [93]	US	Program staff	Cancer control	No	No	Included
Exner-Cortens et al. 2021 [94]	Canada	Teachers and community facilitators	Domestic violence	Yes	No	None
Fazelipour & Cunningham 2019 [95]	Australia, Canada, New Zealand	Multiple	Multiple	No	No	Primary
Feinberg et al. 2008 [96]	US - PA	Coalition members, Program staff	Youth problem behaviors and positive youth development	No	No	None
Fernández et al. 2014 [97]	US - Multiple	Multiple	Cancer control	Yes	No	Included
Flaspohler et al. 2008 [98]	Broadly applicable	Not specified	Multiple	No	No	None
Florin et al. 2012 [99]	US - RI	Program staff	Substance abuse prevention	Yes	No	None
Gandelman et al. 2006 [100]	US	Program staff	HIV prevention	Yes	No	Included
Genat et al. 2016 [101]	Australia	Program staff	Nutrition	No	No	Primary
Gregory et al. 2012 [102]	US - MD	CBO staff	Multiple	No	No	Primary
Haggerty et al. 2017 [103]	US - WA	CBO staff	Positive youth development	No	No	Included
Hannon et al. 2010 [104]	US - Multiple	Coalition members, Program staff	Cancer control	Yes	No	Included
Harshbarger et al. 2006	US	CBO staff	HIV prevention	No	No	Primary
Hawe et al. 1997 [105]	Broadly applicable	Program staff	Not specified	Yes	No	None
Haynes et al. 2014 [106]	US - GA	Multiple	Cancer control	No	No	Primary
Homel et al. 2015 [107]	Australia	Not specified	Crime prevention	No	No	Primary
Honeycutt et al. 2012 [108]	US - GA	Multiple	Nutrition	No	No	Primary
House et al. 2017 [38]	US - Multiple	Program staff	Pregnancy prevention	Yes	Yes	Primary
Hunter et al. 2009 [109]	US - Multiple	Program staff	Substance abuse prevention	Yes	No	Primary
Katz & Wandersman 2016 [26]	Multiple countries	Not specified	Not specified	No	No	None
Kegeles & Rebchook 2005 [110]	US - Multiple	Multiple	HIV prevention	No	No	Primary
Kegeles et al. 2015 [111]	US - Multiple	CBO staff	HIV prevention	No	No	Primary
Kelly et al. 2000 [112]	US - Multiple	CBO staff	HIV prevention	No	No	Included
Kietzman et al. 2019 [113]	US - CA	CBO staff	Multiple	No	No	Primary
Leeman et al. 2015 [25]	Multiple countries	CBO staff	Not specified	Yes	No	None
Leeman et al. 2017 [114]	Multiple countries	CBO staff	Not specified	No	No	None
Leyva et al. 2017 [115]	US - MA	FBO staff, CBO staff	Cancer control	Yes	No	Primary
MacGregor et al. 2013 [116]	Canada	Multiple	Youth violence prevention	Yes	No	Included
MacLean et al. 2003 [117]	Canada	Multiple	Cardiovascular disease	Yes	No	None
Mainor et al. 2018 [118]	US - NC, OR	Program staff	General health promotion	No	No	None
Martinez et al. 2014 [119]	US - PR	CBO staff	Multiple	Yes	No	Primary
Matheson et al. 2020 [120]	New Zealand	Not specified	Multiple	Yes	No	Primary
Miller et al. 2012 [121]	US - MI	CBO staff	Strengthening families for youth with incarcerated parents	No	No	Primary
Mitchell et al. 2002 [122]	US - Multiple	CBO staff	General health promotion	No	No	None
Mueller et al. 2017 [123]	US - Multiple	CBO staff	Pregnancy prevention	No	No	None
Napoles et al. 2013 [124]	US	CBO staff	General health promotion	No	No	Primary
Nargiso et al. 2013 [39]	US - RI	Coalition members	Substance abuse prevention	Yes	Yes	None
Nu'Man et al. 2007 [125]	US	Program staff	HIV prevention	Yes	No	Included
Owczarak 2012 [126]	US - WI	CBO staff	HIV prevention	No	No	Included
Palinkas et al. 2020 [40]	US	Program staff	Mental health, substance abuse prevention	Yes	Yes	None
Peterson et al. 2015 [127]	US - WI	Multiple	Preventing falls among older adults	No	No	None
Pettman et al. 2013 [41]	Australia	Multiple	General health promotion	Yes	Yes	None
Porteny et al. 2020 [128]	US - MA, NY, FL, PR	Program staff	Mental and physical disability prevention	Yes	Yes	Primary
Ramanadhan et al. 2012 [129]	US - MA	CBO staff	Cancer control	No	No	Primary
Ramanadhan et al. 2017 [130]	US - MA	CBO staff	Cancer control	Yes	No	Primary
Ramanadhan et al. 2021 [24]	US - MA	Program staff	General health promotion	No	No	Primary
Roeseler et al. 2011 [131]	US - CA	Multiple	Tobacco control	No	No	Included
Sauaia et al. 2016 [132]	US - CO	Program staff	General health promotion	Yes	No	None
Schoenberg et al. 2021 [133]	US - KY	Program staff	General health promotion	No	No	Primary
Serrano et al. 2020 [134]	Worldwide	Program staff	Not specified	Yes	Yes	None
Sherman & Steiner 2018 [135]	US - MI	CBO staff	Dementia	No	No	None
Veniegas et al. 2009 [136]	US - CA	CBO staff	HIV prevention	Yes	No	Included
Villaruel et al. 2010 [137]	US - AZ, CO, MI	CBO staff	HIV prevention	No	No	Primary
Whitaker et al. 2021 [138]	US - GA	Program staff	Mental health	No	No	Primary
Wilcox et al. 2013 [139]	US	Multiple	Healthy aging	No	No	None
Williams et al. 2019 [140]	US - Multiple	Program staff	Chronic disease prevention	No	No	Primary
Wingfield et al. 2012 [141]	US - GA, NC, SC	FBO staff, CBO staff	Cancer control	No	No	Primary
Yost et al. 2016 [142]	Canada	Multiple	General health promotion	Yes	No	None

CBO = community-based organization; FBO = faith-based organization.

As seen in Table 1, the included studies were published between 1997 and 2021. About half (47%) were published between 1997 and 2014 and the remainder from 2015 to August 2021. A total of 80 were based in the US, 9 were from other high-income countries, 2 explicitly referenced findings in low- and middle-income countries, and 8 did not specify.

### Question 1: How Did Researchers Define Capacity in the Context of CBO Practitioners using EBIs?

Of the 99 articles, 47 defined capacity explicitly (47%), another 31 defined it indirectly (31%), and 21 did not define it at all (21%). Of those that offered direct or indirect definitions, 34 concepts were described, with an average of 3.3 per article. Common concepts included practitioner-level attributes, for example, knowledge and skills, organization-level attributes, for example, leadership and fiscal resources, and system-level attributes, for example, partnerships and informal systems. Among the concepts that were infrequently mentioned, a few related to the broader functioning of groups, communities, or the larger political environment. Overall, 162 concepts (64% of total) were at the practitioner level, 80 (30%) were at the organization level, and 14 (5%) were at the system-level attributes. Concepts mentioned five or more times are presented in Table 2.

**Table 2.** Concepts that appeared in five or more articles, among the 78 studies that offered explicit or indirect definitions of capacity, ordered by decreasing frequency

Concept	Number of articles	Percent
Skills (e.g., for actions needed to use EBIs)	51	65%
Knowledge/expertise (e.g., information about the program)	42	54%
Resources (e.g., constraints or supports on action)	25	32%
Attitudes (e.g., stance on using EBIs)	14	18%
Motivation (e.g., drive to seek EBIs)	12	15%
Self-efficacy/confidence (e.g., a sense that the implementer can take the needed action)	11	14%
Implementation behaviors (e.g., conducting a step in the EBI)	11	14%
Ability (e.g., being capable of implementation)	11	14%
Infrastructure (e.g., formal systems in the organization)	7	9%
Sufficient workforce (e.g., the number and type of needed staff)	7	9%
Leadership (e.g., ability to generate enthusiasm for the EBI)	7	9%
Social networks (e.g., connections among implementers)	7	9%
Organization culture/support (e.g., perceived interest in EBIs at the organization level)	6	8%
Technical/technology (e.g., necessary hardware and software)	5	6%
Readiness (e.g., willingness to address the issue at hand)	5	6%

EBI = evidence-based intervention.

**Fig. 3.**
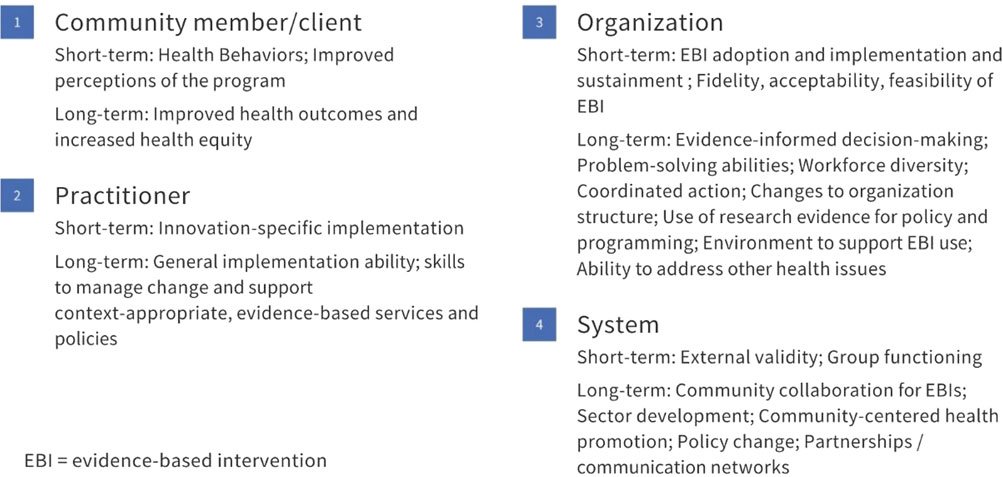
Range of outcomes linked to capacity-building activities (*n* = 99 articles).

We also examined how researchers linked practitioner capacity and capacity-building efforts to key outcomes at multiple levels and across short- and long-term timeframes (Fig. 3).

### Question 2: To What Extent Did Quantitative Studies Measure Capacity, and to What Extent were Psychometric Data Provided?

A total of 57 articles (57%) included quantitative analytic components, and of those, 48 (82%) measured capacity and 11 (23%) offered psychometric data for the capacity measures. The foci and types of psychometric data presented are summarized below.

1. Acosta and colleagues [30] used a combination of a Getting to Outcomes approach and the Consolidated Framework for Implementation Research [31,32] in this study of positive youth development. They defined practitioner prevention capacity in terms of perceived efficacy (ability to complete necessary tasks on one’s own) and behaviors (conducting the necessary implementation tasks, both related to the approach broadly and the intervention specifically. They offered reliability data for each core capacity scale and drew on previously utilized scales.
2. Allen and colleagues [33] conducted a survey that emphasized the importance of skills, availability of skilled staff, organizational supports, and use of research evidence before and after receiving training on evidence-based decision-making. The team scored the perceived importance of each of the ten key skills and the availability of staff members with that skill. Additionally, measures included frequency of using research evidence and work unit and agency expectations and supports for evidence-based decision-making. Finally, a list of steps taken to enhance capacity for evidence-based decision-making was utilized. The measures were validated through five rounds of review by an expert panel, cognitive testing with former state chronic disease directors, and test-retest reliability assessment with state health department staff.
3. Brock and colleagues [34] examined the capacity for a community advisory board (including CBO representatives) to implement an evidence-based obesity program using participatory processes. They used a 63-item survey to capture 13 domains, including capacity efforts (decision-making, conflict resolution, communication, problem assessment, group roles, and resources); capacity outcomes (trust, leadership, participation and influence, collective efficacy); and sustainability outcomes (sustainability, accomplishments, and community power). They reported reliability data for the survey items.
4. Brown and colleagues [35] described measures as part of a protocol for a hybrid, Type 3 cluster-randomized trial examining coalition and prevention program support through technical assistance. Their measure of coalition capacity included cohesion (e.g., sense of unity and trust) and efficiency (e.g., focus and work ethic) for internal team processes.
5. Chinman and colleagues [31] conducted a study based on the Getting to Outcomes framework. For the capacity assessment, they use 23 items to measure self-efficacy (in terms of how much help would be needed) for Getting to Outcomes activities (e.g., conducting a needs assessment). They conducted a factor analysis and assessed the internal consistency reliability of this scale. A separate set of 16 items examined attitudes towards steps of the program process, for example, conducting a formal evaluation. They conducted a factor analysis and calculated internal consistency reliability.
6. Chinman and colleagues [36] conducted a study with the Getting to Outcomes framework and examined prevention capacity as knowledge and skills. The Knowledge Score averaged seven items and examined how much help the respondent would need to carry out a given prevention activity, for example, supporting program sustainability. Internal consistency reliability data were presented. The Skills Score averaged six items and assessed respondents’ frequency of engaging in the prevention activities; internal consistency reliability data were presented.
7. Chinman and colleagues [37] conducted a trial drawing on the Getting to Outcomes framework and key capacity measures focused on efficacy. A five-item efficacy scale focused on respondents’ comfort with engaging in program activities related to asset development. The second efficacy scale focused on comfort implementing the 10-step Getting to Outcomes process. Internal consistency reliability was reported for both scales.
8. House and colleagues [38] drew on the Getting to Outcomes framework and assessed change in capacity for program partners to use EBIs. Relevant items focused on knowledge and confidence in using the Getting to Outcomes process for EBI implementation. Scale reliability data were presented.
9. Nargiso and colleagues [39] examined general capacity of a prevention-focused coalition grounded in the Systems Prevention Framework. Coalitions rated themselves on a 5-point scale for ten items across five domains of capacity: mobilization, structure, task leadership, cohesion, and planning/implementation. They also had an overall coalition capacity score which was a standardized average across the scores. Experts also rated the coalitions regarding leadership, turnover, meetings, visibility, and technological capacity. Inter-rater reliability between participants and experts was calculated. Additionally, the team measured innovation-specific capacity. Experts rated the understanding, partnerships, knowledge of local decision-making related to policy, membership support, and quality of strategic plan. Once more, inter-rater reliability between participants and experts was calculated.
10. Palinkas and colleagues [40] created a measurement for program sustainment that includes a section on “infrastructure and capacity to support sustainment.” Seven items address relevant concepts and data for inter-item reliability, convergent validity, and discriminant validity were presented.
11. Pettman and colleagues [41] measured capacity in terms of implementation behaviors, knowledge, confidence, and attitudes. Although they did not provide psychometric data in the report, they reported using adapted versions of previously validated items.

### Question 3: To What Extent were Studies Focused on Health Equity?

Of the 99 studies, 40 focused exclusively on populations experiencing inequities (40%), 22 included those populations (22%), and 37 did not focus on populations experiencing inequities (37%). As shown in Table 3, the most commonly studied populations included Hispanics/Latinos, African Americans, populations described in the article as “underserved” or low-income, and LGBTQ + populations. We note that the reference to underserved populations did not always include a description of how that was operationalized. Several other priority populations were only represented by one or a small number of studies, for example, people living in rural areas or with disabilities.

**Table 3.** Populations of focus as described in reviewed studies, with some studies addressing the needs of multiple populations (n = 99 articles)

Population	Number
Hispanic/Latino	18
African American	16
Underserved (no specifics provided)	21
Low-income	15
Lesbian, gay, bisexual, transgender, queer (LGBTQ+)	10
Racial/ethnic minorities (no specifics provided)	9
Native American	5
Unhoused	3
Rural	4
Asian or Pacific Islander	1
Incarcerated	1
Aboriginal	1
People with disabilities	2

## Discussion

This scoping review used a comprehensive search strategy to examine how the capacity for EBI use in CBOs is defined and measured. Broadly, our work highlights the need for those addressing capacity-building for EBI use in CBOs to 1) be explicit about models and definitions of capacity-building as implementation strategies, 2) specify expected impacts and outcomes across multiple levels, 3) develop and use validated measures for quantitative work, and 4) integrate equity considerations into the conceptualization and measurement of capacity-building efforts.

First, our results emphasize the need for researchers to be more explicit about their definitions of capacity as a target and capacity-building as a means to support implementation. We found that fewer than half of the articles reviewed offered an explicit definition of capacity. Core concepts covered in definitions centered on practitioner-level attributes, including skills, knowledge, and self-efficacy, though these were not always defined either. At the same time, discussions of practitioner capacity also included organization- and system-level attributes. The variation illustrates the lack of consensus in the field regarding the core dimensions of practitioner capacity [10,42]. Understanding capacity-building efforts as implementation strategies may help prompt reporting that includes details about the involved actors, actions, targets of action, temporality/ordering, dose, expected outcomes, and justification for selection [43].

**Fig. 4.**
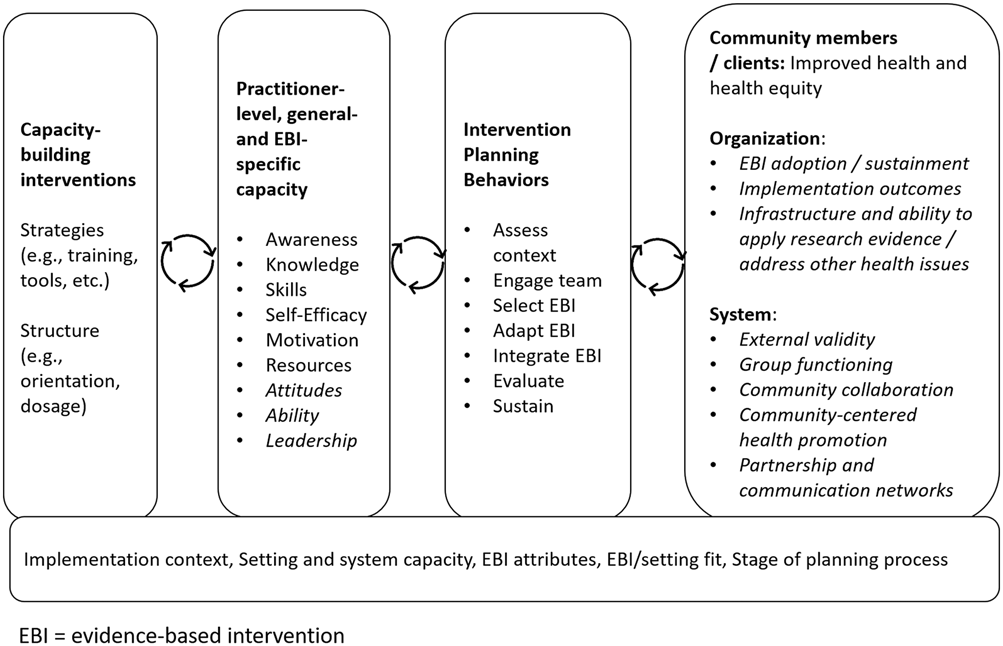
Model of practitioner-level capacity-building, with extensions from review in italics.

In terms of expected impact, the overall takeaway was that *capacity-building is a long-term, dynamic, system-oriented process that transforms resources into short- and long-term change at multiple levels*. Expected impacts ranged from community member/client and practitioner outcomes to organization- and system-level change, echoing other recent reviews of capacity-building [19]. In the context of an outcomes model, such as the Proctor model [44], we might think of short-term impacts of capacity-building as driving implementation outcomes and longer-term outcomes that include a system’s increased ability to utilize research evidence and address new challenges [45]. Viewing capacity-building in the context of professional development prompts the addition of evaluation not only of practitioner skills, knowledge, etc., but also attitudes towards EBIs, job satisfaction and tenure, and other essential supports for EBI delivery in community settings [46]. As summarized in Fig. 4, the review offers a number of extensions to both the dimensions of capacity that warrant further attention as well as to the organization- and system-level outcomes that may result.

The results also highlight a need to improve the use and reporting of validated measures for quantitative assessments. While most quantitative studies measured capacity (48 of 57), only 11 (or 23%) offered psychometric data for these measures. This relates to a broader gap in implementation science highlighted by Lewis and Dorsey, that too few measures have psychometric data, most measures are not applied in different contexts or for different populations, and there are no minimal reporting standards for measures [47]. By increasing the testing of capacity measures for reliability and predictive validity, researchers can address gaps identified through this and previous reviews [20,48]. Other useful potential additions to the literature include identifying “gold standard” measures, determining how and when to measure capacity, gathering data from multiple levels and dynamic systems, and capturing change over time [49]. There is a particular opportunity for implementation scientists to ensure that reporting offers a detailed description of context related to the multiple levels involved in capacity-building, going beyond the required elements to expand on information central to advancing health equity [50–52].

Last, we saw that several studies addressed health inequities, with 62 of the 99 studies focusing or including populations experiencing health inequities. Our work and the broader literature emphasize supporting CBOs in EBI delivery to address health inequities [24,53,54]. At the same time, almost all of the studies that specified a location were grounded in high-income countries. Given that capacity-building is intended to be quite context-specific, this suggests an important gap in the peer-reviewed literature. Stakeholders and researchers in low- and middle-income countries have highlighted gaps in the availability, depth and breadth, support, and local customization based on in-country expertise of capacity-building interventions for EBI use [55,56]. As these gaps are addressed, it may be useful to draw on recent advances in implementation science frameworks that provide guidance on how to operationalize the incorporation of equity goals into implementation planning [57–60].

As with any study, we must ground our findings in the context of a set of limitations. First, we coded data from peer-reviewed articles, many of which had strict word limits. Thus, an activity may have taken place (e.g., validation of a measure) separately from article content. Second, the review focused exclusively on peer-reviewed literature. We are aware of many capacity-building initiatives undertaken by national and international organizations that would not have been included based on our search parameters. Third, we did not examine the details of qualitative assessments of capacity in this analysis but will do so in future work. Finally, although we attempted to build a comprehensive search strategy, we may not have found all of the relevant articles in the field. We tried to reduce this risk by relying on the expertise of a professional librarian. At the same time, several strengths outweigh these weaknesses. First, to our knowledge, this is the first comprehensive review of capacity-building measures for CBOs. Given the importance of CBOs for EBI delivery in support of health equity, this is a significant contribution. Second, we used duplicated screening and coding processes throughout to maintain rigor. Finally, the experience of the team with implementation science, health equity, and CBOs allowed for thoughtful consideration of the research questions and also the interpretation of results.

As measures for capacity among CBOs are strengthened, it will be critical to ensure that the definitions and models resonate with implementers and supporting systems. This may prompt the addition or broadening of some conceptualizations. As noted by Trickett, capacity-building has typically focused on building support for a given research-based resource, but if the goal is sustained use of research evidence, evaluations should also question how this work builds towards other goals in practice and community settings [61]. Through clear specification of capacity-building implementation strategies, use of validated measures for multi-level outcomes, and an intentional equity frame, we can develop high-impact supports for CBO practitioners, a set of critical institutions for addressing health inequities.

## Supplementary material

For supplementary material accompanying this paper visit https://doi.org/10.1017/cts.2022.426. click here to view supplementary material
