# Supplementary material for: Measuring capacity to use evidence-based interventions in community-based organizations: A comprehensive, scoping review
Source: J Clin Transl Sci. 2022 Jul 11;6(1):e92. doi: 10.1017/cts.2022.426 (PMC9389281; doi:10.1017/cts.2022.426)
Supplement: Supplementary file 1 [file ctssup.zip › S2059866122004265sup002.docx]

**Supplemental File 2**

Search terms for scoping review.

Medline/PubMed (National Library of Medicine, NCBI)

("Community Health Workers"[Mesh] OR "Community Health Services"[Mesh:NoExp] OR "Health Promotion"[Mesh:NoExp] OR "Organizations, Nonprofit"[Mesh:NoExp] OR "Health Education"[Mesh:NoExp] OR "Patient Education as Topic"[Mesh] OR "Consumer Health Information"[Mesh] OR community-based[tiab] OR community health[tiab] OR consumer health[tiab] OR health education[tiab] OR health promotion[tiab] OR Lady health worker*[tiab] OR Lay health worker*[tiab] OR Village health worker*[tiab] OR local organization*[tiab] OR non-clinical[tiab] OR non profit*[tiab] OR nonprofit*[tiab] OR prevention support[tiab] OR community organization*[tiab] OR “Public Health Practice”[Mesh:noexp] OR public health practic*[tiab])

AND

("Evidence-Based Practice"[Mesh:noexp] OR "Implementation Science"[Mesh] OR evidence based[tiab] OR evidence informed[tiab] OR effective intervention*[tiab] OR knowledge translation[tiab] OR implementation science[tiab] OR practice-based evidence [tiab])

AND

("Capacity Building"[Mesh] OR "Professional Competence"[Mesh:NoExp] OR "Staff Development"[Mesh] OR capacity[tiab] OR competencies[tiab] OR skills[tiab] OR work force[tiab] OR workforce[tiab] OR professional development[tiab] OR staff[tiab] OR practitioners[tiab] OR knowledge broker*[tiab])

Web of Science, Core Collection (Clarivate)

Editions: Science Citation Index Expanded; Social Sciences Citation Index

Advanced Search

TS=("community-based" OR "community health" OR "consumer health" OR "health education" OR "health promotion" OR "Lady health worker*" OR "Lay health worker*" OR "Village health worker*" OR "local organization*" OR "non-clinical" OR "non profit*" OR "nonprofit*" OR "prevention support" OR "community organization*" OR "public health practic*")

AND

TS=("evidence based" OR "evidence informed" OR "effective intervention*" OR "knowledge translation" OR "implementation science" OR "practice-based evidence")

AND

TS=(capacity OR competencies OR skills OR "work force" OR workforce OR "professional development" OR staff OR practitioners OR "knowledge broker*")

Global Health Database (C.A.B. International, Ebsco)

Remove: Apply equivalent subjects

Document Type: Journal Article

1)

DE "medical auxiliaries" OR DE "community health services" OR DE "health services" DE "health promotion" OR DE "health education" OR DE "patient education"

2)

IN TITLE OR ABSTRACT OR SUBJECTS

"community-based" OR "community health" OR "consumer health" OR "health education" OR "health promotion" OR "Lady health worker*" OR "Lay health worker*" OR "Village health worker*" OR "local organization*" OR "non-clinical" OR "non profit*" OR "nonprofit*" OR "prevention support" OR "community organization*" OR "public health practic*"

3)

IN TITLE OR ABSTRACT OR SUBJECTS

"evidence based" OR "evidence informed" OR "effective intervention*" OR "knowledge translation" OR "implementation science" OR "practice-based evidence"

4)

DE "professional competence" OR DE "career development" OR DE "job skills" OR DE "continuing education" OR DE "workers" OR DE "health care workers"

5)

IN TITLE OR ABSTRACT OR SUBJECTS

capacity OR competencies OR skills OR "work force" OR workforce OR "professional development" OR staff OR practitioners OR "knowledge broker*"

(1 OR 2) AND 3 AND (4 OR 5)
